# Deletion of Robo4 worsens neuroinflammation in a mouse model of Alzheimer's disease

**DOI:** 10.14814/phy2.70950

**Published:** 2026-06-04

**Authors:** Abigail E. Cullen, Nick R. Winder, Byron Lee, Sahana Krishna Kumaran, Nayantara Arora, Julia R. Wolf, Grant D. Henson, Randall L. Woltjer, Ashley E. Walker

**Affiliations:** ^1^ Department of Human Physiology University of Oregon Eugene Oregon USA; ^2^ Department of Pathology and Laboratory Medicine Oregon Health & Science Univ. Portland Oregon USA

**Keywords:** Alzheimer's disease, neuroinflammation, Robo4

## Abstract

Declines in vascular integrity are potential contributors to Alzheimer's disease (AD) as these result in increased blood–brain barrier permeability and, consequently, accelerate neuroinflammation and cognitive impairment. Roundabout guidance receptor 4 (Robo4) is primarily expressed in endothelial cells and stabilizes the vasculature, thereby potentially protecting the brain in AD. To study the effect of Robo4 on neuroinflammation and cognitive function in the context of AD, we compared *Robo4* knockout and wild‐type mice crossed with mice with and without AD mutations (APP/tau) from heterogeneous age and sex groups. We found that knockout of Robo4 led to greater astrocyte activation, as demonstrated by GFAP content, but this effect depended on the brain region studied. The knockout of *Robo4* also led to greater activated microglia, as assessed by Iba1 content, but only in the presence of AD‐related mutations. We found that AD mutations, but not Robo4 mutations, were associated with cognitive dysfunction, as measured by a nest‐building test. Lastly, there was a non‐statistically significant trend toward Robo4 deletion being associated with greater arterial stiffness. In summary, these results demonstrate that *Robo4* impacts neuroinflammation and arterial stiffness; however, the impact on neuroinflammation is dependent on the presence/absence of AD‐related mutations and the brain region examined.

## 1 INTRODUCTION

It is becoming increasingly evident that vascular dysfunction is a contributor to Alzheimer's disease (AD) and other dementias (Sweeney et al., 2018). Featuring prominently in this vascular dysfunction is a decrease in vascular integrity (Sweeney et al., 2018). In the brain, a decrease in vascular integrity leads to an increase in the permeability of the blood–brain barrier (BBB) that allows neurotoxic substances, inflammatory cells, and pathogens to enter the brain (Sweeney et al., 2018). As such, greater BBB permeability is associated with increases in neuroinflammation, and neuroinflammation is further associated with the cognitive impairments and neuropathology that embody AD (Heneka et al., 2015). Indeed, greater BBB permeability is an early marker for cognitive decline (Nation et al., 2019). However, the role of vascular integrity in AD is somewhat contradictory. While decreased integrity and increased BBB permeability have negative consequences, there is also evidence that increased signaling for vascular permeability has benefits. Central to these beneficial effects is vascular endothelial growth factor (VEGF), which increases permeability but is also associated with angiogenesis and positive outcomes in AD (Garcia et al., 2014; Hohman et al., 2015). Given the unclear role of vascular integrity in AD, a better understanding of these signaling pathways in the context of AD is needed.

The roundabout family of transmembrane receptors has a role in vascular integrity. Roundabouts are best known for their role in axon guidance, and their primary ligands are Slit proteins (London & Li, 2011). Roundabout guidance receptor 4 (Robo4) is primarily expressed in endothelial cells and stabilizes the vasculature (Jones et al., 2008). Slit2, a ligand for Robo4, inhibits VEGF signaling in endothelial cells (Jones et al., 2008) and inhibits the vascular permeabilizing effects of pro‐inflammatory cytokines, effects that are dependent on Robo4 (London et al., 2010). Slit2 is also known to inhibit endothelial cell migration and angiogenesis (London et al., 2010). In summary, Robo4/Slit2 signaling increases vascular stability, and a decrease in this signaling increases vascular permeability.

Reductions in Robo4/Slit2 signaling are implicated in several brain‐related impairments, such as cerebral endothelial cell dysfunction, increased BBB permeability, and neuroinflammation. For example, cerebral endothelial cells from old female mice exhibit lower Robo4 expression than their old male counterparts. It is suggested that this lower endothelial cell Robo4 expression increases the vulnerability to stressors, such as susceptibility to the cellular senescence caused by iron overload (Noh et al., 2023). Additionally, brain endothelial cells from diabetic rats have lower Robo4 expression than cells from nondiabetic rats, and this decreased Robo4 expression is associated with detrimental excessive neovascularization (Abdelsaid et al., 2017). Relatedly, Slit2 overexpression is associated with better cognitive performance and better paravascular clearance in mice (He et al., 2020; Li et al., 2018). Related to neuroinflammation, Slit2 overexpression is associated with fewer reactive astrocytes (He et al., 2020; Li et al., 2018) and fewer microglia in mice that were given cerebral microinfarcts (He et al., 2020). Thus, the Robo4 (and Slit2) pathway is known to have beneficial effects on the brain. However, the effects of Robo4 directly on neuroinflammation have not been studied, nor has Robo4 been studied in the context of AD. It is unknown whether AD‐related pathology (Aβ and tau) modulates the effects of Robo4.

To study the effects of Robo4 in the context of AD, we examined mice with *Robo4* knockout (KO) crossed with mice with AD mutations (APPswe and tauP301L). We hypothesized that *Robo4* KO mice would have greater neuroinflammation and worse cognitive function compared to Robo4 wild‐type (WT) mice. We further hypothesized that there would be an interaction between *Robo4* and AD mutations, such that the *Robo4* KO × AD+ group would have worse neuroinflammation and cognitive function compared with both the *Robo4* KO × AD‐ and *Robo4* WT × AD+ groups. We found that *Robo4* deletion leads to greater neuroinflammation. In agreement with our hypothesis, the presence of AD‐related mutations exacerbated the impact of *Robo4* deletion, particularly for microglia content.

## 2 METHODS

### 2.1 Animals

To study the effects of Robo4 in the context of AD, we generated a cross between AD (APPswe and tauP301L) mice (Oddo et al., 2003) with *Robo4* knockout mice (Jones et al., 2008) to generate four different genotypes: AD‐ × *Robo4* WT, AD‐ × *Robo4* KO, AD+ × *Robo4* WT, and AD+ × *Robo4* KO. *Robo4* KO mice were obtained from Dr. Dean Li's laboratory at the University of Utah. The AD mice had a knock‐in of the amyloid precursor protein Swedish mutation and the tauP301L mutation, obtained from the Jackson Laboratory. Male and female mice were studied at young (6–10 mo), middle‐age (11–14 mo), or old (19–26 mo) ages. We found no age or sex differences for any outcomes within each genotype, and thus, we combined the age and sex groups for each genotype. The exception to this is that we found an age effect for Iba1 in the entorhinal cortex, but this outcome did not show genotype effects. After collecting behavior measurements, mice were euthanized by thoracotomy and exsanguination under isoflurane, and perfused with saline. Following saline, mice were perfused with 4% paraformaldehyde to preserve cerebral tissue. All mice were housed in an animal care facility on a 12/12 h light–dark cycle at 24°C. Animals had access to ad libitum food (Lab Diet, PicoLab Rodent Diet 20, 5053) and water. All animal procedures conformed to the *Guide to the Care and Use of Laboratory Animals* (8th version) and were approved by the Institutional Animal Care and Use Committee at the University of Oregon.

### 2.2 Aortic stiffness

We measured aortic stiffness using pulse wave velocity. Mice were anesthetized using 3% isoflurane in 100% oxygen and then placed in the supine position on a temperature‐controlled surgical pad at 37°C (Indus Instruments, Webster, TX, USA). ECG was recorded throughout the procedure and two Doppler Transducers were positioned at the aortic arch and abdominal aorta to collect waveforms simultaneously. After recording 3 ~ 10 s intervals of the Doppler signal, the distance between the two probes was measured. To analyze the difference in pulse arrival times, the Doppler Signal Processing Workstation program (Indus Industries, Webster, TX, USA) was used. Two blinded researchers analyzed each file independently, and inter‐observation confidence intervals were measured; those over 15% were reanalyzed by a third researcher. PWV was calculated by dividing the distance (in cm) by ejection time recorded between the two Doppler Transducers.

### 2.3 Cognitive function

Nest Building: To test for changes in instinctual behavior, we conducted a nest‐building test. Mice were singly housed during the dark cycle for 12 h in a cage containing a cotton nestlet and no other enrichment. At the end of the 12 h, the cotton nestlet was scored against a preset scale from 0–5, with 0 indicating an untouched nestlet and 5 indicating a fully built nest (Deacon, 2012).

Open Field: To test for changes in anxiety levels we conducted the open‐field test, as previously described (Reeve et al., 2023). Briefly, mice were placed in an enclosed, empty arena (40 cm by 40 cm) and allowed to explore for 10 min; during this time a video was recorded using Ethovision software (version 12, Noldus, Leesburg, VA, USA). Analysis of open field was conducted with Ethovision and exploration criteria were defined as the amount of time the mouse spent in a predetermined inner square (20 cm by 20 cm) of the area versus the outer perimeter of the arena, where mice that spent more time in the inner square were considered to have less anxiety.

### 2.4 Histology

After paraformaldehyde perfusion, brains were incubated in 4% paraformaldehyde for 48 h and then transferred to distilled water for longer term storage. Brains were embedded in paraffin before being cut at 5 μm thickness. Immunohistochemical staining was conducted as described previously (Woltjer et al., 2015), using primary antibodies for Iba1 (1:5000, ProteinTech, 10904‐1‐AP), GFAP (1:500, ProteinTech, 16825‐1‐AP), and vimentin (1:500, ProteinTech, 10366‐1‐AP), 4G8 (1:5000, Biolegend, SIG‐39200), a rabbit secondary antibody (1:1000, Vector Laboratories, BA‐1000‐1.5), and DAB (1:1000, 3,3′‐diaminobenzidine, AFG Bioscience, 91‐95‐2). Analysis of histology was conducted by first imaging the regions of interest (hippocampus (CA1), entorhinal cortex, and thalamus) using a Leica DMi1 microscope with a 20x objective. The Image J software (NIH, Bethesda, MD, USA) was used for analysis of percent positive area. No positive staining for Aβ (4G8) was observed in any sample, and therefore, we were unable to quantify this marker.

### 2.5 Statistical analysis

Statistical analyses were performed with GraphPad Prism 10.0. A one‐way analysis of variance (ANOVA) between different ages (young, middle, and old age) and between sexes within each genotype was conducted initially to determine whether groups could be combined. For all outcomes, the resulting *p*‐values for sex and age within genotype groups was *p* > 0.05, and thus, statistical analysis was performed between genotype groups containing all ages and sexes. Outliers were determined to be values with *Z*‐scores outside the range of −2.0 to 2.0 and were removed from the data set. Outliers that were removed include: 2 GFAP hippocampus (1 AD‐ × *Robo4* KO, 1 AD+ × *Robo4* KO), 2 GFAP thalamus (1 AD‐ × *Robo4* KO, 1 AD+ × *Robo4* WT), 4 GFAP entorhinal corte× (1 each group), 3 Iba1 hippocampus (1 AD‐ × *Robo4* WT, 1 AD‐ × *Robo4* KO, 1 AD+ × *Robo4* KO), 3 Iba1 thalamus (1 AD‐ × *Robo4* WT, 1 AD+ × *Robo4* WT, 1 AD+ × *Robo4* KO), and 3 Iba1 entorhinal cortex (1 AD‐ × *Robo4* WT, 1 AD‐ × *Robo4* WT, 1 AD+ × *Robo4* KO). All groups were normally distributed as assessed by a Shapiro–Wilk test. If normality was not met, data was transformed by taking the square root of each sample. The Shapiro–Wilk test was used again to confirm normality was met if the data was transformed. A two‐way ANOVA (AD genotype × Robo4 genotype) was conducted for all outcomes. In cases of a significant *F* value, post hoc analyses were performed using the Tukey correction for preplanned comparisons. Significance was set at *p* < 0.05 and all values shown are means ± standard deviation.

## 3 RESULTS

To establish our model, we measured *Robo4* gene expression in the hippocampus and found significantly less expression of *Robo4* in our KO mice (Figure S1A, *p* = 0.007). We also measured Slit2 gene expression and found no difference in expression between *Robo4* WT and KO mice (Figure S1B, *p* = 0.76). We did not observe amyloid‐β (Aβ) plaques in brain tissue from any mice (data not shown).

### 3.1 Robo4 deletion leads to astrocyte activation in AD mice

**FIGURE 1:**
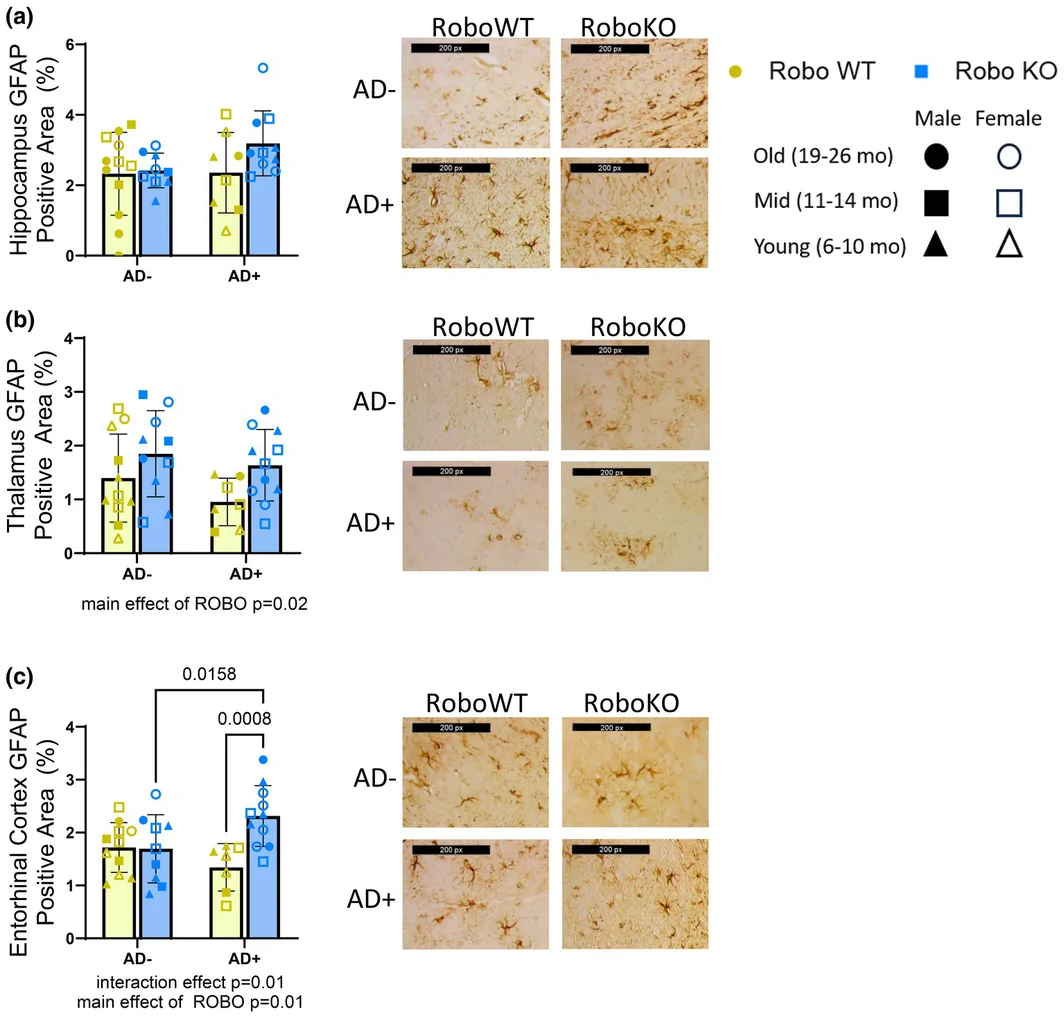
Astrocyte content is influenced by *Robo4* and AD genes. GFAP quantified by immunohistochemistry in the (a) hippocampus, (b) thalamus, and (c) entorhinal cortex for Robo4 wildtype (WT) or knockout (KO) mice that had mutant Alzheimer's disease (AD) genes present (+) or absent (−) (*n* = 7–12/group). Neither *Robo4* nor AD genes caused significant differences in GFAP content in the hippocampus. Conversely, there was a main effect of *Robo4* genes on GFAP content in the thalamus and entorhinal cortex (*p* = 0.01 and 0.007, respectively). There was an interaction effect of AD and *Robo4* and a main effect of *Robo4* in the entorhinal cortex (*p* = 0.01 and 0.01, respectively). Representative images to the right. Values are mean ± *SD*.

GFAP and vimentin was used to identify the activation of astrocytes. In the hippocampus, we observed no main or interaction effects of AD and *Robo4* genotype on GFAP expression, indicating that in the hippocampus, neither genotype influences the activation of astrocytes (Figure 1a, all *p* > 0.05).

In the thalamus, there was a main effect of *Robo4* genotype on GFAP expression (Figure 1b, *p* = 0.02). In contrast, we observed a main effect of AD on vimentin expression in the thalamus (Figure S2B, *p* = 0.02). These results suggest that the effects of AD and *Robo4* on astrocytes in the thalamus depend on the marker measured.

In the entorhinal cortex, we observed similar trends for GFAP as in the thalamus with the addition of an interaction effect between AD and *Robo4* (Figure 1c, *p* = 0.01). There was a main effect of *Robo4* genotype on GFAP expression in the entorhinal cortex (Figure 1c, *p* = 0.01). In the *Robo4* mice, mice carrying AD genes had significantly more GFAP expression compared to mice without AD genes (Figure 1c, *p* = 0.01). In the AD mice, *Robo4* KO mice had more GFAP expression compared with the *Robo4* WT mice (Figure 1c, *p* = 0.0008).

For GFAP outcomes, we found no effect of age or sex with any genotype group (*p* > 0.05). The data for vimentin expression in the hippocampus and entorhinal cortex were not normally distributed after transformation and were therefore not analyzed. The transformed data can be found in Figure S2A,C. When separated by age and sex, we saw similar trends for astrocyte content.

### 3.2 Robo4 deletion leads to lower microglia content in AD mice

**FIGURE 2:**
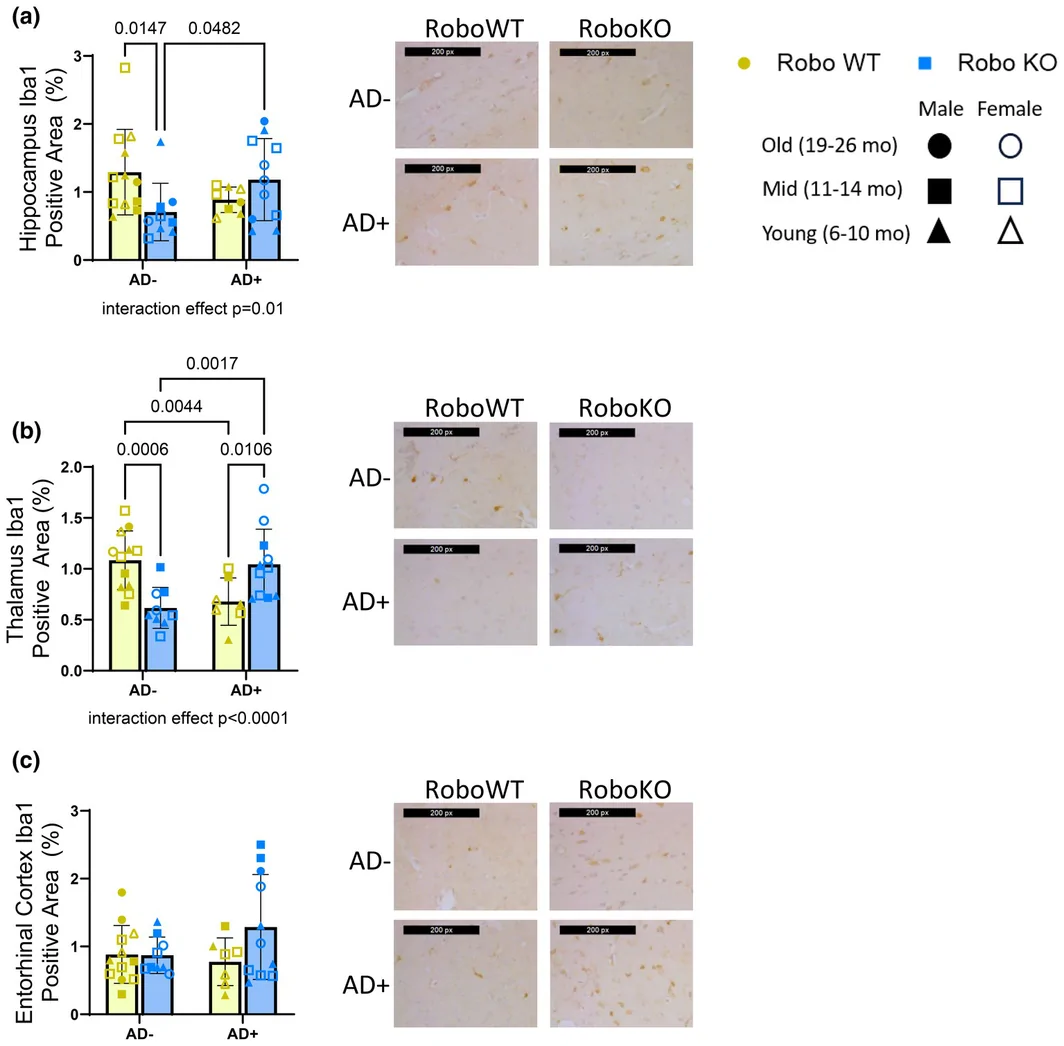
Microglia content is influenced by both *Robo4* and AD genes. Iba1 quantified by immunohistochemistry in the (a) hippocampus, (b) thalamus, and (c) entorhinal cortex for Robo4 wildtype (WT) or knockout (KO) mice that had mutant Alzheimer's disease (AD) genes present (+) or absent (−). There was an interaction effect in the hippocampus and thalamus for Iba1 expression (*p* = 0.01 and <0.0001, respectively). There was no effect of *Robo4* or AD genes on Iba1 expression in the entorhinal cortex. Representative images to the right. Values are mean ± *SD*.

We measured Iba1 in the hippocampus, thalamus, and entorhinal cortex as it identifies microglia. In the hippocampus and thalamus, there was an interaction effect between *Robo4* and AD genotype on Iba1 expression (Figure 2a,b, *p* = 0.01 and *p* < 0.0001, respectively). There was a trend for lower Iba1 expression in the hippocampus of *Robo4* KO animals compared to their *Robo4* WT counterparts (Figure 2a, *p* = 0.04). There was significantly less Iba1 expression in AD WT × *Robo4* KO compared to AD WT × *Robo4* WT (Figure 2a, *p* = 0.0147). There was significantly reduced Iba1 expression in the thalamus of *Robo4* WT × AD animals compared to *Robo4* WT (Figure 2b, *p* = 0.004). In *Robo4* KO, Iba1 expression was significantly reduced compared to their *Robo4* KO × AD counterparts (Figure 2b, *p* = 0.0017). In the absence of AD genes, Iba1 expression was significantly lower in *Robo4* KO mice than in *Robo4* WT (Figure 2b, *p* = 0.0006). Conversely, in the presence of AD genes, *Robo4* KO mice showed significantly higher Iba1 expression than *Robo4* WT (Figure 2b, *p* = 0.01). In contrast to the thalamus, there were no additional differences detected in the hippocampus (Figure 2a, all *p* > 0.05). While there were interaction effects in the hippocampus and thalamus, there were no significant interactions or main effects for Iba1 in the entorhinal cortex (Figure 2c, all *p* > 0.05). For Iba1 outcomes, we found no effect of age or sex with any genotype group (*p* > 0.05). These results indicate that *Robo4* has a differential impact on microglial content depending on the presence of mutant AD genes and location within the brain. When separated by age and sex, we saw similar trends for microglial content.

### 3.3 Robo4 deletion does not affect nest‐building or anxiety

**FIGURE 3:**
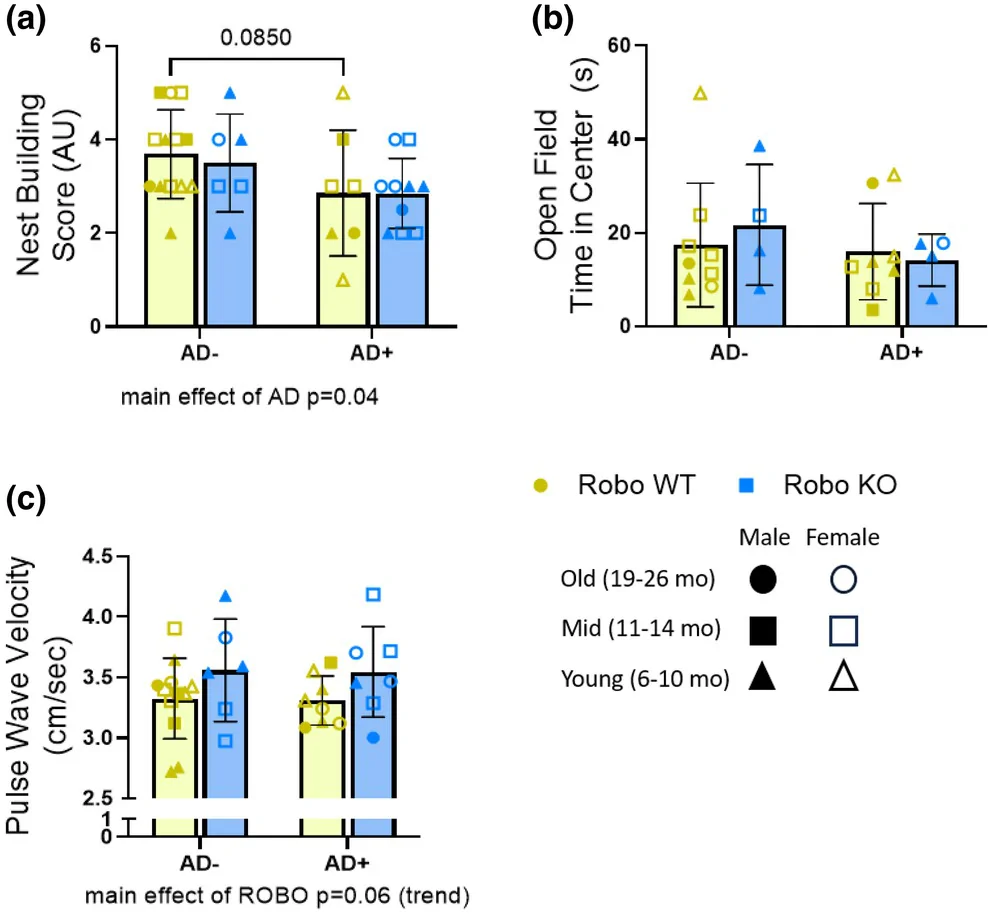
*Robo4* and AD genes influence cognitive and cardiovascular function. We assessed instinctual behavior by nesting building score (a), anxiety during an open field test (b), and aortic stiffness by pulse wave velocity (c) in Robo4 wild‐type (WT) or knockout (KO) mice that had mutant Alzheimer's disease (AD) genes present (+) or absent (−). Instinctual behavior was influenced by AD genes, with AD+ mice having a worse nest‐building score (*p* = 0.04). *Robo4* and AD genes did not influence anxiety or aortic stiffness; however, there was a trend for *Robo4* KO mice to have a higher aortic pulse wave velocity (*p* = 0.06). Representative images to the right. Values are mean ± *SD*.

Instinctual behavior, measured by the nest‐building test, was significantly impacted by AD genotype, where the presence of AD mutations led to a significant reduction in nest quality (Figure 3a, main effect: *p* = 0.04). There was no impact of *Robo4* genotype on nest building (Figure 3a, *p* > 0.05). Anxiety, measured by the open field test, was not influenced by *Robo4* or AD genotypes (Figure 3b, *p* > 0.05).

### 3.4 Trend for greater aortic stiffness with Robo4 deletion

There was no effect of AD genotype on aortic PWV (Figure 3c, *p* > 0.05); however, there was a trend for a main effect of the *Robo4* genotype (Figure 3c, *p* = 0.06), such that *Robo4* KO mice tend to have higher aortic PWV.

## 4 DISCUSSION

In this study, we demonstrated the impact of *Robo4*, in combination with AD‐related mutations, on neuroinflammation, cognitive function, and arterial stiffness. We found that the knockout of *Robo4* led to greater astrocyte activation, as demonstrated by GFAP content. However, the GFAP results did not align with vimentin, another astrocyte marker, which demonstrated an increase in activated astrocytes due to AD mutations, but not Robo4 knockout. We also found that the knockout of *Robo4* led to greater activated microglia, as assessed by Iba1, but only in the presence of AD‐related mutations. We found that AD mutations, but not *Robo4*, were associated with cognitive dysfunction by nest building. Lastly, *Robo4* deletion was associated with greater arterial stiffness, but this trend did not reach statistical significance. In summary, these results demonstrate that Robo4 impacts neuroinflammation and arterial stiffness; however, the impact on neuroinflammation is dependent on the presence/absence of AD‐related mutations and the brain region examined.

### 4.1 Robo4, neuroinflammation, and BBB permeability

Robo4 prevents neuroinflammation, as demonstrated by our study and others. In our study, we find that *Robo4* deletion leads to greater reactive astrocytes and microglia, at least in the thalamus and cortex. These results are in line with previous studies reporting that overexpression of Slit2, the primary Robo4 ligand, leads to fewer reactive astrocytes (He et al., 2020; Li et al., 2018). Previous studies have found that Slit2 impacts neuroinflammation in the hippocampus and cerebral cortex (Li et al., 2018), while our study found effects only in the cortex and thalamus. In our study, the presence of AD mutations appears to amplify the impact of *Robo4* deletion on neuroinflammation. Similar to our findings, others have found that the benefits of Robo/Slit are amplified in disease conditions. For example, it was previously found that Slit2 overexpression does not impact microglial activation in middle‐aged control (sham) mice (He et al., 2020). However, in mice who were given cerebral microinfarcts, Slit2 overexpression led to less microglia activation compared with mice without Slit2 overexpression (He et al., 2020). Thus, it appears that decreases in Slit/Robo signaling lead to neuroinflammation, but to have a substantial impact, other stressors need to be present. This suggests that perturbations in Slit/Robo signaling may have minimal impact in young, healthy conditions but may increase their impact in the presence of other stressors.

The mechanism underlying Robo4's impact on neuroinflammation is likely due to the modulation of BBB permeability. A leaky BBB has the potential to allow pro‐inflammatory molecules and cells from the blood to enter the brain, thus triggering neuroinflammation (Sweeney et al., 2018). Robo4 is known to regulate endothelial cell interactions, impacting permeability and sprouting in established blood vessels (Jones et al., 2008). In this way, Robo4/Slit2 signaling leads to increased stability of cerebral endothelial cells and a less permeable BBB. The stabilization of the BBB by Robo4 is most likely mediated by inhibition of VEGF signaling and reducing the pro‐permeability effects of inflammatory factors (Jones et al., 2008). For example, IL‐1β, TNF, and LPS induce endothelial barrier permeability, but this is inhibited by Slit2 in a Robo4‐dependent manner (London et al., 2010). However, in the absence of VEGF activation, Robo/Slit2 modulation has minimal effects (Jones et al., 2008). As such, the most striking effects of Robo4 on endothelial barrier permeability will occur in the presence of a stressor. Similarly, it was previously shown that *Robo4* deletion induces endothelial cell senescence, but only when an additional stressor (in this case iron overload) was present (Noh et al., 2023). Thus, the absence of Robo4 can lead to endothelial barrier dysfunction and increased BBB permeability, and this is most likely to occur in the presence of pro‐angiogenic stimuli, such as aging or brain/vascular injury. This, in part, may explain why we found regional‐specific differences in neuroinflammation in the *Robo4* KO mice.

It should be mentioned that the pro‐stability effects of Robo/Slit signaling are not entirely consistent in the literature. One previous study found that Slit2 overexpression in wild‐type mice leads to greater BBB permeability (Li et al., 2015); however, this study was performed in otherwise healthy mice, and thus, the finding may be due to the lack of a pro‐permeability stimulus. In addition, the activation of other Slit or Robo isoforms can lead to vascular instability; for example, Slit binding to Robo1 promotes endothelial cell motility, an opposite effect of Robo4 (Marlow et al., 2010). Further, Slit3 can activate Robo4, and this causes increased angiogenesis, the opposite effect of Slit2 (Zhang et al., 2009). Similarly, Slit2 can be both pro‐ and anti‐angiogenic, with the pro‐angiogenic effects being mediated by mTOR, while the anti‐angiogenic effects require ephrin‐A1 (Dunaway et al., 2011). We also previously demonstrated that Robo4 has anti‐vasodilatory effects, specifically impaired endothelium‐dependent vasodilation (Phuong et al., 2019). This impaired vasodilation may be due to the inhibition of nitric oxide production from VEGF pathways with Robo4 activation. These effects could potentially lead to reduced cerebral blood flow or neurovascular coupling. Thus, while Robo4 appears important for maintaining the BBB and preventing neuroinflammation during pro‐angiogenic conditions, this may be context‐specific and the impact of Robo4 on vasodilation should be considered.

### 4.2 Robo4, instinctual behavior, and anxiety

The effects of Robo4 signaling on neurocognitive function are inconsistent in the literature. In our study, we did not find that *Robo4* deletion impacted nest building, while the AD‐related mutations led to the expected worse performance. In contrast, previous studies have found that Slit2 overexpression, which activates Robo4, leads to better performance on the Morris Water Maze in both middle‐aged mice and after brain injury (He et al., 2020; Li et al., 2018). The difference between our study and this previous study could be that the Morris Water Maze test is more sensitive to changes in hippocampal‐dependent memory than nest building. Adding further inconsistencies, another study found that Slit2 overexpression worsens cognitive function, but this study was conducted in otherwise healthy mice (Li et al., 2015). Thus, the effects of Robo4 on memory are inconsistent in the literature and need further investigation.

### 4.3 Robo4 and arterial stiffness

We find that *Robo4* deletion was associated with a higher aortic stiffness, but this trend did not reach statistical significance. While the effects of Robo4 on arterial stiffness have not been previously studied, it is known that Robo4 impacts the extracellular matrix. For example, *Robo4* knockdown increases MMP‐9 activity (Cai et al., 2015). However, seemingly contradictory to our finding, the knockdown of Slit2 leads to decreased collagen expression in cardiac tissue through Robo1 (Liu et al., 2021). The tissue of study (vascular vs. cardiac) and Robo isoform may explain the differences between our study and previous findings. Therefore, further studies are needed to understand the mechanisms by which Robo4 may affect arterial stiffness.

## 5 LIMITATIONS

There are a few limitations to this study that should be considered. First, the *Robo4* deletion was not specific to cerebral endothelial cells. Therefore, the deletion of *Robo4* from endothelial cells outside the brain could impact the outcomes, potentially by systemic actions. Second, the *Robo4* deletion was constitutive and thus was present during development. However, previous studies have shown no differences in vascular structure observed during development in these mice (Jones et al., 2008). Third, we pooled a range of ages for our analysis. While age and sex comparisons revealed no statistical differences for all outcomes (with the exception of Iba1 in the entorhinal cortex), our sample size is small for each age × sex group within each genotype. As such, we do not fully have the power to detect if age and sex differences are present in our sample. The effects of age on Iba1 in the entorhinal cortex do not impact our conclusions because we found no genotype effects for this outcome. Fourth, the AD mice did not display a phenotype that would be expected of an AD mouse model, such as the presence of Aβ plaques and greater neuroinflammation. However, most AD mouse models include mutations in presenilin‐1 (*PS1*) or multiple *APP* mutations. The mice in this study lacked *PS1* mutations and contained only the *APP* ^*swe*^ mutation, and furthermore, due to the crossing with Robo, these mice were also *APP* ^*swe*^ heterozygotes. Thus, the mice in this study constitute a milder AD model than in previous studies. Lastly, due to a limited number of mice, we were unable to perform functional BBB permeability studies; therefore, we cannot definitively say if the effects we see are due to increased endothelial cell permeability.

## 6 CONCLUSIONS

The primary findings from this study are that *Robo4* deletion leads to greater neuroinflammation. The presence of AD‐related mutations further strengthened the impact of *Robo4* deletion, particularly for microglia activation. These results suggest that Robo4 is important for squelching neuroinflammation in the face of AD. Our findings align with previous studies that show a benefit of Robo4 in maintaining a healthy cerebral vasculature, particularly in the face of injury and/or inflammation. These studies add further evidence in support of targeting the Slit2/Robo4 pathway to limit the damage during AD. However, future studies are required to identify the mechanisms by which Robo4 modulates neuroinflammation.

## AUTHOR CONTRIBUTIONS

**Abigail E. Cullen:** Data curation; formal analysis; investigation; methodology. **Nick R. Winder:** Investigation; methodology. **Byron Lee:** Investigation; methodology. **Sahana Krishna Kumaran:** Investigation; methodology. **Nayantara Arora:** Investigation; methodology. **Julia R. Wolf:** Investigation; methodology. **Grant D. Henson:** Investigation; methodology. **Randall L. Woltjer:** Conceptualization; formal analysis; investigation; methodology; resources. **Ashley E. Walker:** Conceptualization; data curation; formal analysis; funding acquisition; investigation; methodology; project administration; resources; supervision.

## FUNDING INFORMATION

This work was supported by the National Institutes of Health (NIH) Grant R01AG064016 (to AEW), TL1TR002371 (to AEC), and the Oregon Alzheimer's Disease Research Center P30AG066518 (to RLW).

## ETHICS STATEMENT

All animal procedures conform to the Guide to the Care and Use of Laboratory Animals (8th edition, revised 2011) and were approved by the Institutional Animal Care and Use Committees at the University of Oregon (protocol #AUP‐20‐25).

## Supporting information


**Supplemental Figure 1.** Hippocampal gene expression of Robo4 and Slit2. We assessed hippocampal gene expression of both Robo4 (A) and Slit2 (B) in the Robo4 KO mice. There was a significant difference in expression in Robo4 expression (*p* = 0.007), but no significant difference in Slit2 expression (*p* = 0.76).
**Supplemental Figure 2**. AD genes influence vimentin expression in the thalamus. We assessed vimentin expression in the hippocampus (A), thalamus (B), and entorhinal cortex (C). There was a main effect of AD genes in the thalamus. Vimentin expression data from the hippocampus and entorhinal cortex did not pass normality and was not analyzed, data are shown for visual representation. Representative images to the right. Values are mean ± *SD*.

## References

[phy270950-bib-0001] Abdelsaid, M. , Coucha, M. , Hafez, S. , Yasir, A. , Johnson, M. H. , & Ergul, A. (2017). Enhanced VEGF signalling mediates cerebral neovascularisation via downregulation of guidance protein ROBO4 in a rat model of diabetes. Diabetologia, 60, 740–750. 28116460 10.1007/s00125-017-4214-6 PMC5342922

[phy270950-bib-0002] Cai, H. , Liu, W. , Xue, Y. , Shang, X. , Liu, J. , Li, Z. , Wang, P. , Liu, L. , Hu, Y. , & Liu, Y. (2015). Roundabout 4 regulates blood‐tumor barrier permeability through the modulation of ZO‐1, Occludin, and Claudin‐5 expression. Journal of Neuropathology and Experimental Neurology, 74, 25–37. 25470344 10.1097/NEN.0000000000000146

[phy270950-bib-0003] Deacon, R. (2012). Assessing burrowing, nest construction, and hoarding in mice. Journal of Visualized Experiments, 59, e2607. 10.3791/2607 PMC3369766 22258546

[phy270950-bib-0004] Dunaway, C. M. , Hwang, Y. , Lindsley, C. W. , Cook, R. S. , Wu, J. Y. , Boothby, M. , Chen, J. , & Brantley‐Sieders, D. M. (2011). Cooperative signaling between Slit2 and ephrin‐A1 regulates a balance between angiogenesis and angiostasis. Molecular and Cellular Biology, 31, 404–416. 21135133 10.1128/MCB.00667-10 PMC3028625

[phy270950-bib-0005] Garcia, K. , Ornellas, F. , Matsumoto, P. , Patti, C. , Mello, L. , Frussa‐Filho, R. , Han, S. , & Longo, B. (2014). Therapeutic effects of the transplantation of VEGF overexpressing bone marrow mesenchymal stem cells in the hippocampus of murine model of Alzheimer's disease. Frontiers in Aging Neuroscience, 6, Article 30. 24639647 10.3389/fnagi.2014.00030 PMC3945612

[phy270950-bib-0006] He, X.‐F. , Li, G. , Li, L.‐l. , Li, M.‐Y. , Liang, F.‐Y. , Chen, X. , & Hu, X.‐Q. (2020). Overexpression of Slit2 decreases neuronal excitotoxicity, accelerates glymphatic clearance, and improves cognition in a multiple microinfarcts model. Molecular Brain, 13, 135. 33028376 10.1186/s13041-020-00659-5 PMC7542754

[phy270950-bib-0007] Heneka, M. T. , Carson, M. J. , El Khoury, J. , Landreth, G. E. , Brosseron, F. , Feinstein, D. L. , Jacobs, A. H. , Wyss‐Coray, T. , Vitorica, J. , Ransohoff, R. M. , Herrup, K. , Frautschy, S. A. , Finsen, B. , Brown, G. C. , Verkhratsky, A. , Yamanaka, K. , Koistinaho, J. , Latz, E. , Halle, A. , … Kummer, M. P. (2015). Neuroinflammation in Alzheimer's disease. Lancet Neurology, 14, 388–405. 25792098 10.1016/S1474-4422(15)70016-5 PMC5909703

[phy270950-bib-0008] Hohman, T. J. , Bell, S. P. , Jefferson, A. L. , & Alzheimer's Disease Neuroimaging, I . (2015). The role of vascular endothelial growth factor in neurodegeneration and cognitive decline: Exploring interactions with biomarkers of Alzheimer disease. JAMA Neurology, 72, 520–529. 25751166 10.1001/jamaneurol.2014.4761 PMC4428948

[phy270950-bib-0009] Jones, C. A. , London, N. R. , Chen, H. , Park, K. W. , Sauvaget, D. , Stockton, R. A. , Wythe, J. D. , Suh, W. , Larrieu‐Lahargue, F. , Mukouyama, Y. S. , Lindblom, P. , Seth, P. , Frias, A. , Nishiya, N. , Ginsberg, M. H. , Gerhardt, H. , Zhang, K. , & Li, D. Y. (2008). Robo4 stabilizes the vascular network by inhibiting pathologic angiogenesis and endothelial hyperpermeability. Nature Medicine, 14, 448–453. 10.1038/nm1742 PMC2875252 18345009

[phy270950-bib-0010] Li, G. , He, X. , Li, H. , Wu, Y. , Guan, Y. , Liu, S. , Jia, H. , Li, Y. , Wang, L. , Huang, R. , Pei, Z. , Lan, Y. , & Zhang, Y. (2018). Overexpression of Slit2 improves function of the paravascular pathway in the aging mouse brain. International Journal of Molecular Medicine, 42, 1935–1944. 30085336 10.3892/ijmm.2018.3802 PMC6108881

[phy270950-bib-0011] Li, J. C. , Han, L. , Wen, Y. X. , Yang, Y. X. , Li, S. , Li, X. S. , Zhao, C. J. , Wang, T. Y. , Chen, H. , Liu, Y. , Qi, C. L. , He, X. D. , Gu, Q. L. , Ye, Y. X. , Zhang, Y. , Huang, R. , Wu, Y. E. , He, R. R. , Kurihara, H. , … Wang, L. J. (2015). Increased permeability of the blood‐brain barrier and Alzheimer's disease‐like alterations in slit‐2 transgenic mice. Journal of Alzheimer's Disease, 43, 535–548. 10.3233/JAD-141215 25114073

[phy270950-bib-0012] Liu, Y. , Yin, Z. , Xu, X. , Liu, C. , Duan, X. , Song, Q. , Tuo, Y. , Wang, C. , Yang, J. , & Yin, S. (2021). Crosstalk between the activated Slit2‐Robo1 pathway and TGF‐beta1 signalling promotes cardiac fibrosis. ESC Heart Failure, 8, 447–460. 33236535 10.1002/ehf2.13095 PMC7835586

[phy270950-bib-0013] London, N. R. , & Li, D. Y. (2011). Robo4‐dependent slit signaling stabilizes the vasculature during pathologic angiogenesis and cytokine storm. Current Opinion in Hematology, 18, 186–190. 21423011 10.1097/MOH.0b013e328345a4b9 PMC3541019

[phy270950-bib-0014] London, N. R. , Zhu, W. , Bozza, F. A. , Smith, M. C. , Greif, D. M. , Sorensen, L. K. , Chen, L. , Kaminoh, Y. , Chan, A. C. , Passi, S. F. , Smith, M. C. P. , Day, C. W. , Barnard, D. L. , Zimmerman, G. A. , Krasnow, M. A. , & Li, D. Y. (2010). Targeting Robo4‐dependent slit signaling to survive the cytokine storm in sepsis and influenza. Science Translational Medicine, 2, 23ra19. 10.1126/scitranslmed.3000678 PMC2875996 20375003

[phy270950-bib-0015] Marlow, R. , Binnewies, M. , Sorensen, L. K. , Monica, S. D. , Strickland, P. , Forsberg, E. C. , Li, D. Y. , & Hinck, L. (2010). Vascular Robo4 restricts proangiogenic VEGF signaling in breast. Proceedings of the National Academy of Sciences of the United States of America, 107, 10520–10525. 20498081 10.1073/pnas.1001896107 PMC2890778

[phy270950-bib-0016] Nation, D. A. , Sweeney, M. D. , Montagne, A. , Sagare, A. P. , D'Orazio, L. M. , Pachicano, M. , Sepehrband, F. , Nelson, A. R. , Buennagel, D. P. , Harrington, M. G. , D'Orazio, L. M. , Benzinger, T. L. S. , Fagan, A. M. , Ringman, J. M. , Schneider, L. S. , Morris, J. C. , Chui, H. C. , Law, M. , Toga, A. W. , & Zlokovic, B. V. (2019). Blood‐brain barrier breakdown is an early biomarker of human cognitive dysfunction. Nature Medicine, 25, 270–276. 10.1038/s41591-018-0297-y PMC6367058 30643288

[phy270950-bib-0017] Noh, B. , Blasco‐Conesa, M. P. , Rahman, S. M. , Monga, S. , Ritzel, R. , Guzman, G. , Lai, Y. J. , Ganesh, B. P. , Urayama, A. , McCullough, L. D. , & Moruno‐Manchon, J. F. (2023). Iron overload induces cerebral endothelial senescence in aged mice and in primary culture in a sex‐dependent manner. Aging Cell, 22, e13977. 37675802 10.1111/acel.13977 PMC10652299

[phy270950-bib-0018] Oddo, S. , Caccamo, A. , Shepherd, J. D. , Murphy, M. P. , Golde, T. E. , Kayed, R. , Metherate, R. , Mattson, M. P. , Akbari, Y. , & LaFerla, F. M. (2003). Triple‐transgenic model of Alzheimer's disease with plaques and tangles: Intracellular Abeta and synaptic dysfunction. Neuron, 39, 409–421. 12895417 10.1016/s0896-6273(03)00434-3

[phy270950-bib-0019] Phuong, T. T. T. , Walker, A. E. , Henson, G. D. , Machin, D. R. , Li, D. Y. , Donato, A. J. , & Lesniewski, L. A. (2019). Deletion of Robo4 prevents high‐fat diet‐induced adipose artery and systemic metabolic dysfunction. Microcirculation, 26, e12540. 30825241 10.1111/micc.12540 PMC7217325

[phy270950-bib-0020] Reeve, E. H. , Kronquist, E. K. , Wolf, J. R. , Lee, B. , Khurana, A. , Pham, H. , Cullen, A. E. , Peterson, J. A. , Meza, A. , Colton Bramwell, R. , Villasana, L. , Machin, D. R. , Henson, G. D. , & Walker, A. E. (2023). Pyridoxamine treatment ameliorates large artery stiffening and cerebral artery endothelial dysfunction in old mice. Journal of Cerebral Blood Flow and Metabolism, 43, 281–295. 36189840 10.1177/0271678X221130124 PMC9903220

[phy270950-bib-0021] Sweeney, M. D. , Kisler, K. , Montagne, A. , Toga, A. W. , & Zlokovic, B. V. (2018). The role of brain vasculature in neurodegenerative disorders. Nature Neuroscience, 21, 1318–1331. 30250261 10.1038/s41593-018-0234-x PMC6198802

[phy270950-bib-0022] Woltjer, R. L. , Reese, L. C. , Richardson, B. E. , Tran, H. , Green, S. , Pham, T. , Chalupsky, M. , Gabriel, I. , Light, T. , Sanford, L. , Jeong, S. Y. , Hamada, J. , Schwanemann, L. K. , Rogers, C. , Gregory, A. , Hogarth, P. , & Hayflick, S. J. (2015). Pallidal neuronal apolipoprotein E in pantothenate kinase‐associated neurodegeneration recapitulates ischemic injury to the globus pallidus. Molecular Genetics and Metabolism, 116, 289–297. 26547561 10.1016/j.ymgme.2015.10.012 PMC4688119

[phy270950-bib-0023] Zhang, B. , Dietrich, U. M. , Geng, J. G. , Bicknell, R. , Esko, J. D. , & Wang, L. (2009). Repulsive axon guidance molecule Slit3 is a novel angiogenic factor. Blood, 114, 4300–4309. 19741192 10.1182/blood-2008-12-193326 PMC2774558

