# Supplementary material for: Deletion of Robo4 worsens neuroinflammation in a mouse model of Alzheimer's disease
Source: Physiol Rep. 2026 Jun 4;14(11):e70950. doi: 10.14814/phy2.70950 (PMC13581104; doi:10.14814/phy2.70950)
Supplement: Supplementary file 1 [file PHY2-14-e70950-s001.pdf]

Figure 1.

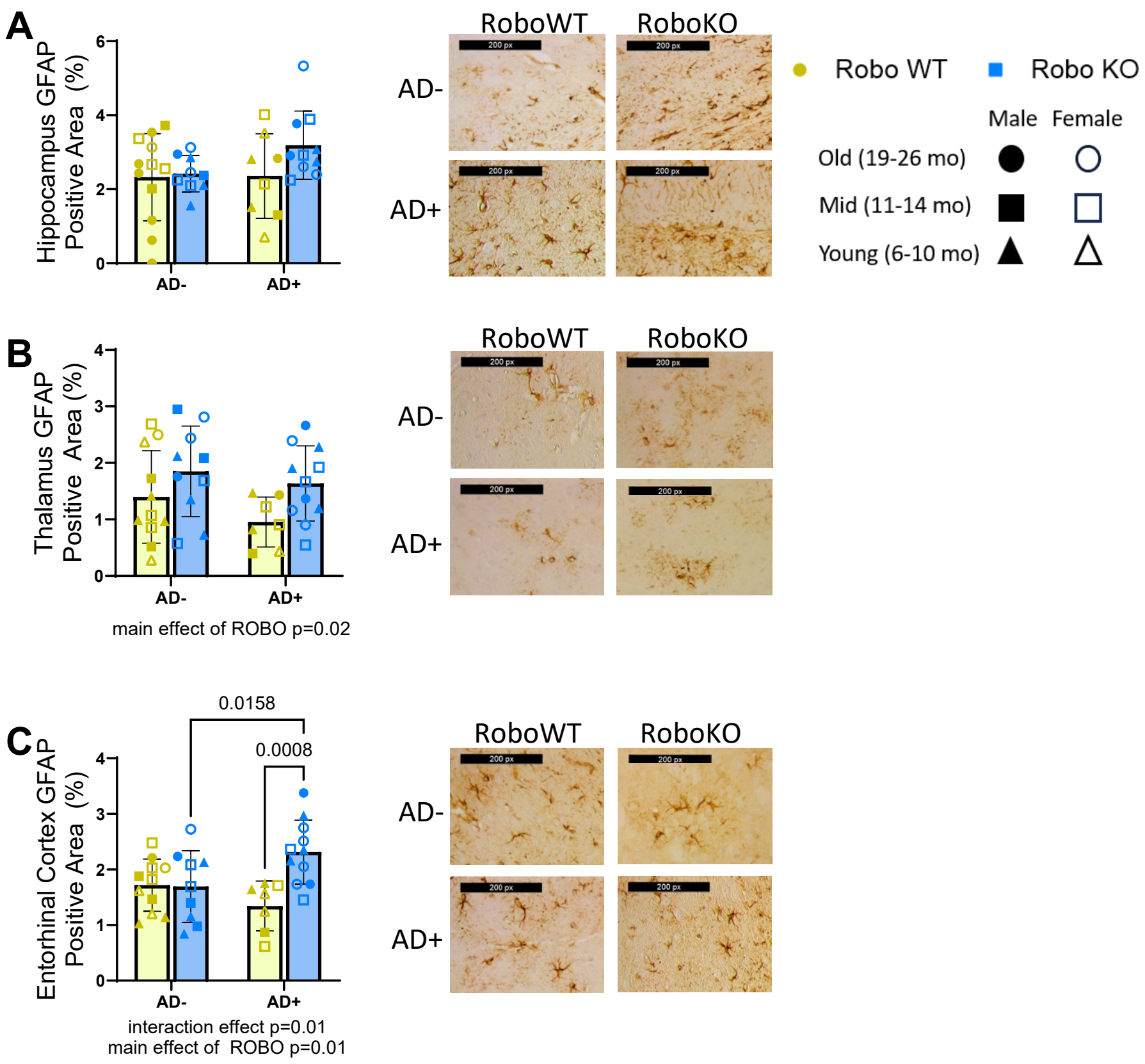
A
Hippocampus GFAP Positive Area (%)
AD-
AD+
RoboWT
RoboKO
AD-
AD+
Robo WT
Robo KO
Male
Female
Old (19-26 mo)
Mid (11-14 mo)
Young (6-10 mo)
B
Thalamus GFAP Positive Area (%)
AD-
AD+
main effect of ROBO p=0.02
RoboWT
RoboKO
AD-
AD+
C
Entorhinal Cortex GFAP Positive Area (%)
0.0158
0.0008
AD-
AD+
interaction effect p=0.01
main effect of ROBO p=0.01
RoboWT
RoboKO
AD-
AD+

Figure 2.

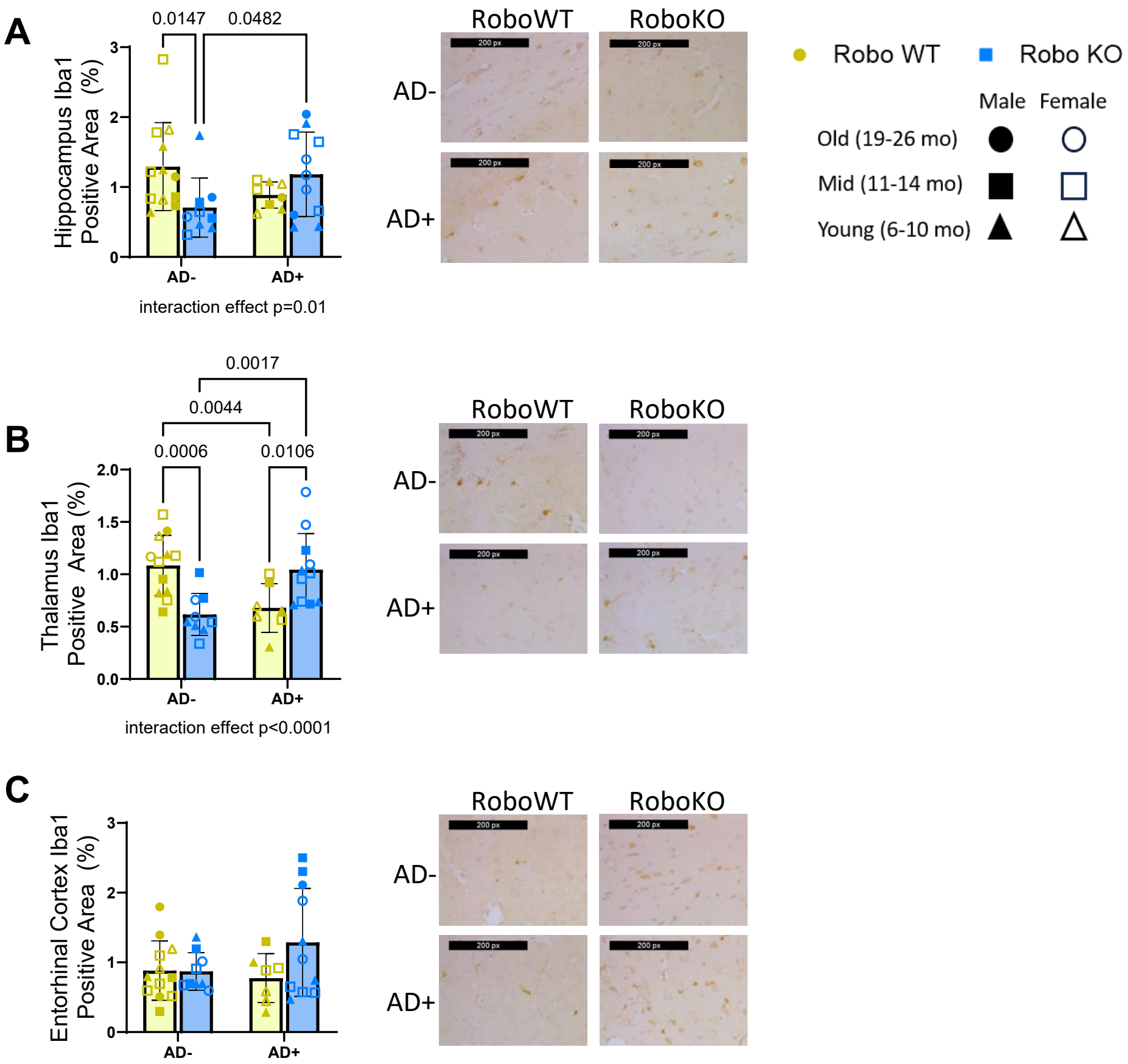

A
0.0147
0.0482
Hippocampus Iba1 Positive Area (%)
AD-
AD+
interaction effect p=0.01
RoboWT
RoboKO
Robo WT
Robo KO
Male
Female
Old (19-26 mo)
Mid (11-14 mo)
Young (6-10 mo)
B
0.0017
0.0044
0.0006
0.0106
Thalamus Iba1 Positive Area (%)
interaction effect p<0.0001
C
Entorhinal Cortex Iba1 Positive Area (%)

Figure 3.

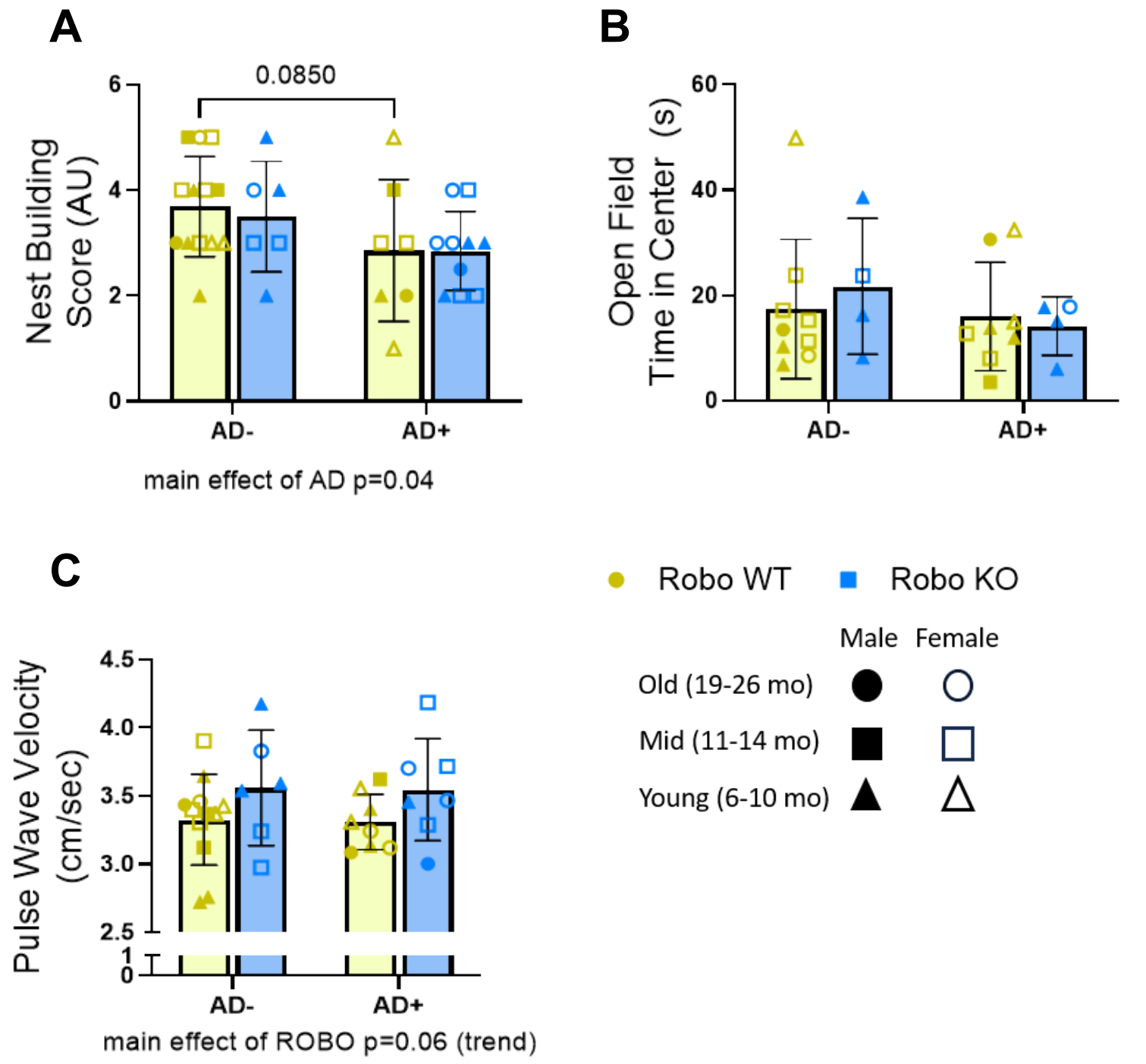
A
B
0.0850
Nest Building Score (AU)
6
4
2
0
AD-
AD+
main effect of AD p=0.04
Open Field Time in Center (s)
60
40
20
0
AD-
AD+
C
Pulse Wave Velocity (cm/sec)
4.5
4.0
3.5
3.0
2.5
1
0
AD-
AD+
main effect of ROBO p=0.06 (trend)
Robo WT
Robo KO
Male
Female
Old (19-26 mo)
Mid (11-14 mo)
Young (6-10 mo)

SuppFig1

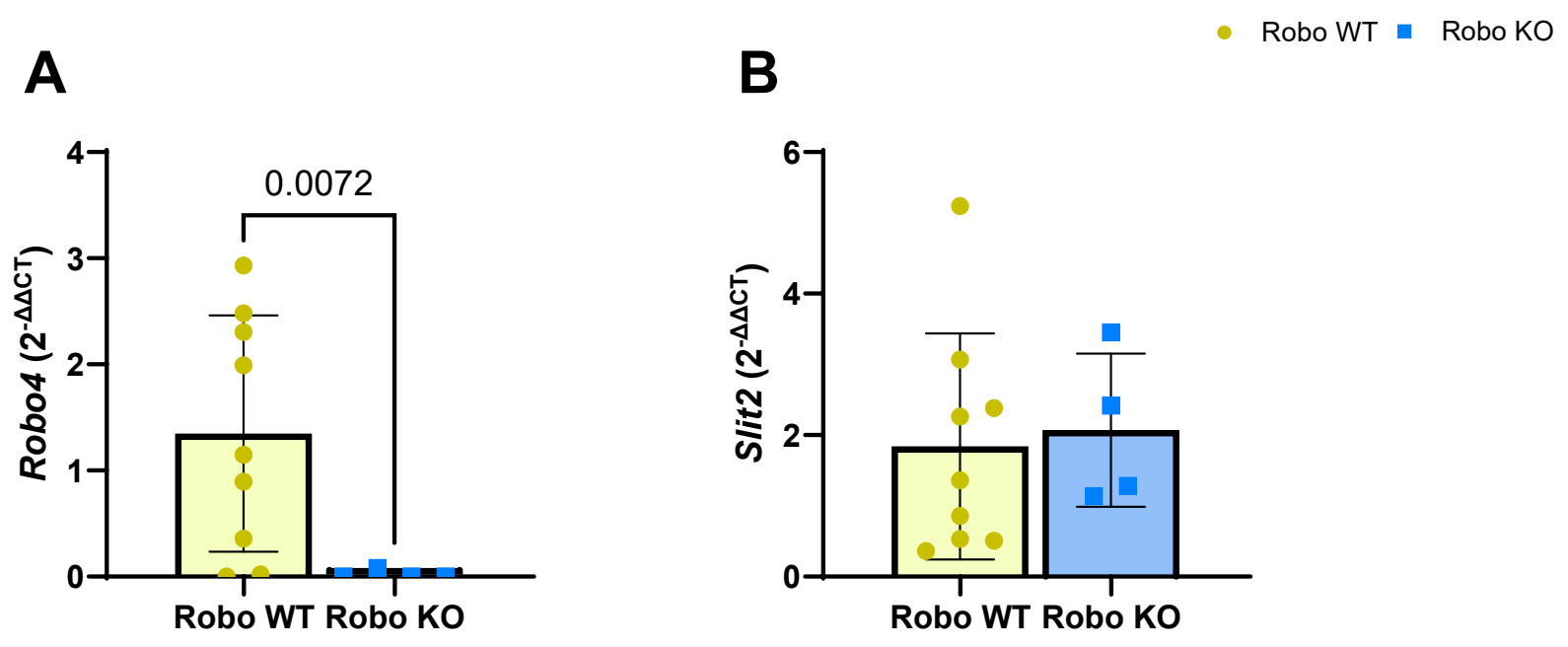
Robo WT
Robo KO
A
B
0.0072
Robo4 (2-ΔΔCT)
Slit2 (2-ΔΔCT)
4
3
2
1
0
6
4
2
0
Robo WT Robo KO
Robo WT Robo KO

SuppFig2

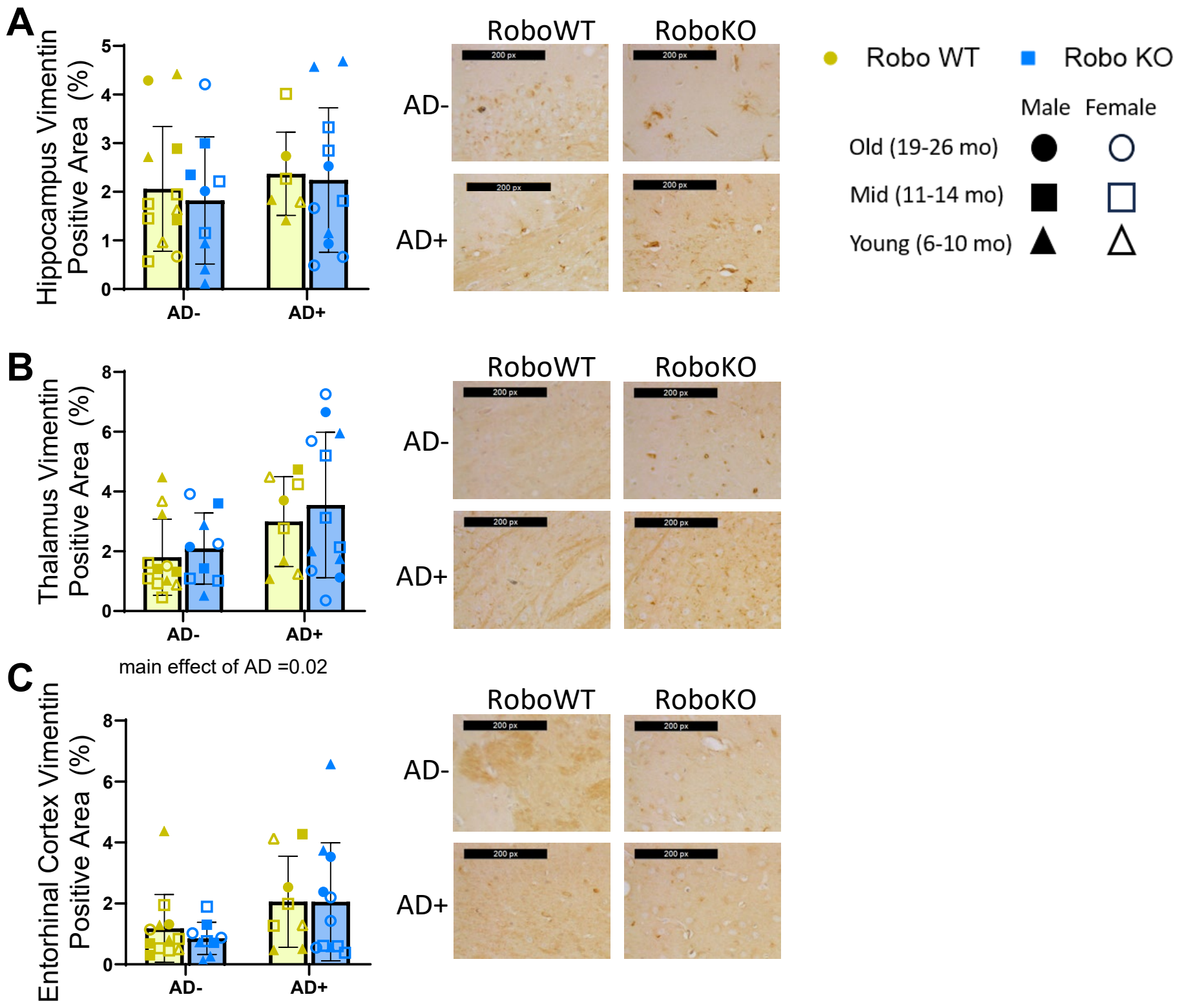

A
Hippocampus Vimentin Positive Area (%)
AD-
AD+
RoboWT
RoboKO
Robo WT
Robo KO
Male
Female
Old (19-26 mo)
Mid (11-14 mo)
Young (6-10 mo)
B
Thalamus Vimentin Positive Area (%)
main effect of AD =0.02
C
Entorhinal Cortex Vimentin Positive Area (%)
